# Seasonal structure of interactions enhances multidimensional stability of mutualistic networks

**DOI:** 10.1098/rspb.2022.0064

**Published:** 2022-09-14

**Authors:** François Duchenne, Rafael O. Wüest, Catherine H. Graham

**Affiliations:** Swiss Federal Institute for Forest, Snow and Landscape Research (WSL), 8903 Birmensdorf, Switzerland

**Keywords:** hummingbird, resilience, community, interaction network, pollination, phenology

## Abstract

Community ecologists have made great advances in understanding how natural communities can be both diverse and stable by studying communities as interaction networks. However, focus has been on interaction networks aggregated over time, neglecting the consequences of the seasonal organization of interactions (hereafter 'seasonal structure') for community stability. Here, we extended previous theoretical findings on the topic in two ways: (i) by integrating empirical seasonal structure of 11 plant–hummingbird communities into dynamic models, and (ii) by tackling multiple facets of network stability together. We show that, in a competition context, seasonal structure enhances community stability by allowing diverse and resilient communities while preserving their robustness to species extinctions. The positive effects of empirical seasonal structure on network stability vanished when using randomized seasonal structures, suggesting that eco-evolutionary dynamics produce stabilizing seasonal structures. We also show that the effects of seasonal structure on community stability are mainly mediated by changes in network structure and productivity, suggesting that the seasonal structure of a community is an important and yet neglected aspect in the diversity–stability and diversity–productivity debates.

## 1. Introduction

Identifying the mechanisms that stabilize communities is a major goal of ecology. During the last decades, mutualistic communities, in which all species involved in an interaction directly benefit from this interaction (e.g. pollination), have been shown to be stabilized by the non-random organization of interactions [1–4]. However, most studies used interaction networks aggregated in time, thus neglecting temporal interaction turnover, despite pioneering studies showing that seasonal structure of interactions, henceforth seasonal structure, is an important determinant of network structure [5,6]. Recent work shows that phenological traits influence mutualistic interactions [7–10], stressing the need to shift from a static view of interaction networks to a dynamic view of these systems [11].

The importance of seasonal structure on network stability has been evaluated based on temporal variation of different facets of stability, including feasibility (i.e. size of the parameter domain in which all species can coexist) [12], resilience (i.e. the inverse of the time to return to the equilibrium after a small perturbation) [12] and robustness (i.e. resistance to species extinction) [13,14]. Generally, networks aggregated over an entire year (overall communities) are decomposed into seasonal sub-communities (e.g. weekly communities) that are studied independently. As a result, most studies focus on seasonal variation in structure and stability rather than how these seasonal dynamics influence the stability of the overall community. A few theoretical studies have shown that seasonal structure of interactions not only creates seasonal variation in network structure and stability but also increases the stability of the overall community, measured as resilience or persistence (i.e. percentage of initial species persisting at equilibrium) [15,16]. This positive effect of seasonal structure on persistence comes from the fact that interactions are not all simultaneous but instead, spread across seasons. These temporal dynamics reduce competition and, at the same time, maintain facilitation among species that share common mutualistic partners at different points in time, resulting in increased network persistence [17]. However, evidence that seasonal structure increases overall network stability is still limited to theoretical work evaluating only one or two stability facets (persistence and resilience). In the real world evolutionary dynamics tend to optimize species fitness and not community persistence [18], hence there is no reason why evolutionary trajectories of species would lead to a seasonal structure that would favour network stability. Thus, additional work is needed to verify that the positive effects of the seasonal structure found in simulated ecological equilibriums [15,17] hold for (i) empirical seasonal structures, which are a result of evolutionary history, and (ii) for different facets of stability, which often provide opposing insights [12].

**Figure 1.**
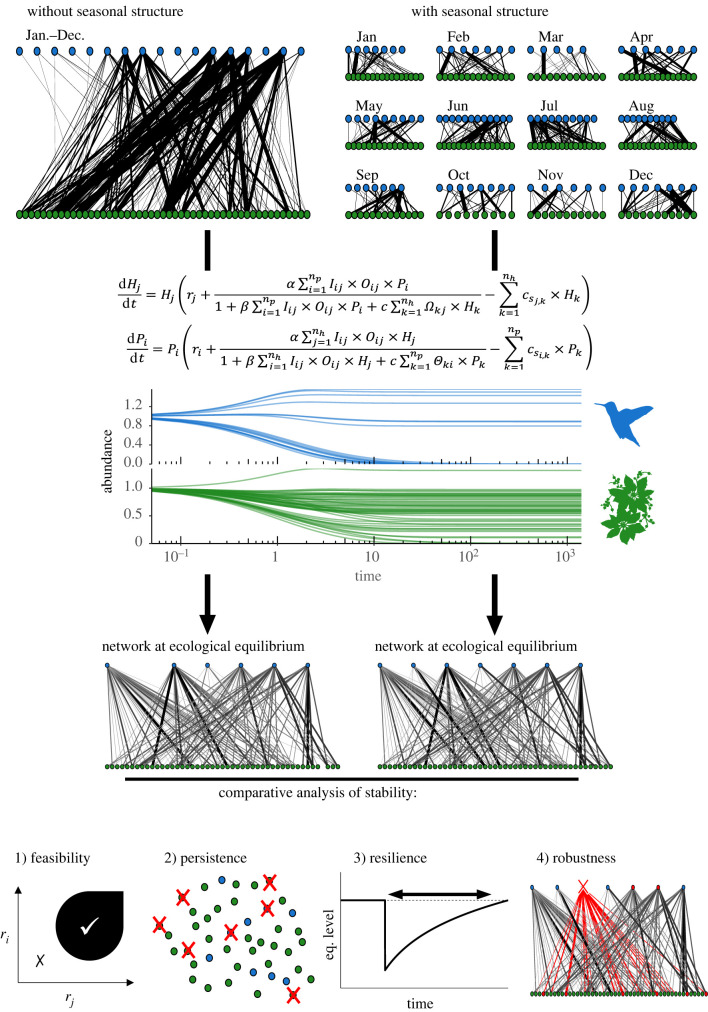
Schematic view of the methods used here. Interaction data, on the top, are considered either by not accounting (left) or accounting for the seasonal structure of interactions (right). These data are used to parametrize a dynamic model (cf. Methods for the details of equations and assumptions), which is solved until an ecological equilibrium is reached (i.e. constant species abundances over time). We then compared four facets of the network stability for simulations without or with seasonal structure: (1) feasibility, percentage of simulations, over a range of growth rates (*r_i_* and *r_j_*), in which equilibrium contains more than 98% of the species present in each community; (2) persistence, percentage of species present at the equilibrium; (3) resilience, the invert of the time to return at equilibrium after a small perturbation; and (4) robustness, percentage of species of a community surviving to the extinction of a given species. We repeated this workflow for each community independently and for a range of competition and mutualistic strengths. (Online version in colour.)

To study the consequences of seasonal structure of interactions for network stability, we anchored theoretical approaches in data, using dynamic models parametrized with empirical seasonal structures from 11 plant–hummingbird interaction networks (see 'Methods'; electronic supplementary material, figures S1 and S2). Since mutualistic interactions tend to increase persistence while competition tends to decrease it [19,20], we expected that the outcome of the seasonal structure on species persistence would depend on the strength of competition and mutualism. The fact that interactions are spread over the year has two important consequences: (i) it decreases the availability of mutualistic partners, because species can interact only with species that co-occur in time, and (ii) it decreases competition for mutualistic partners among species from the same guild, because species compete for a mutualistic partner only if they interact with it at the same time. Thus, our model included competition among species from the same guild which interact with common mutualistic partners at the same time. We performed simulations for different combinations of mutualism strength (*α*) and competition strength (*c*), for each of the 11 communities. Here, mutualism strength (*α*), also referred to as γ [19,21], is defined as the positive effect of mutualistic interactions on *per capita* growth rates of species involved in these interactions, thus describing the efficiency of mutualistic interactions in providing resources for pollinators and pollination for plants. When *α* = 0, mutualistic interactions do not affect populations' growth, whereas greater *α* values mean greater positive effect of mutualistic interactions on *per capita* growth rates. Since *α* is embedded in a complex functional response (see 'Methods'), the final effects of mutualistic interactions on *per capita* growth rates will also depend on competition for mutualistic partners (*c*). To determine the influence of seasonal structure on stability, we divided simulations in two sets: one neglecting the seasonal structure, setting all phenological overlap to one (cf. Methods) and another one accounting for the seasonal structure, using empirical phenological overlaps. We compared network stability between the two sets of simulations at equilibrium, with or without seasonal structure (figure 1).

To address the fact that stability is a multidimensional concept [22,23], we evaluated four complementary facets: (1) the feasibility, i.e. percentage of simulations, over a range of plant and hummingbird growth rates, in which at least 98% of the initial species persist at equilibrium (see 'Methods'), (2) the persistence, i.e. percentage of species surviving at equilibrium, which is strongly linked to network feasibility, (3) the resilience, i.e. invert of the time to return to the equilibrium after a small perturbation and (4) the robustness, i.e. percentage of species of the community surviving after the extinction of a given species (until a next equilibrium is reached; see 'Methods').

## 2. Results

**Figure 2.**
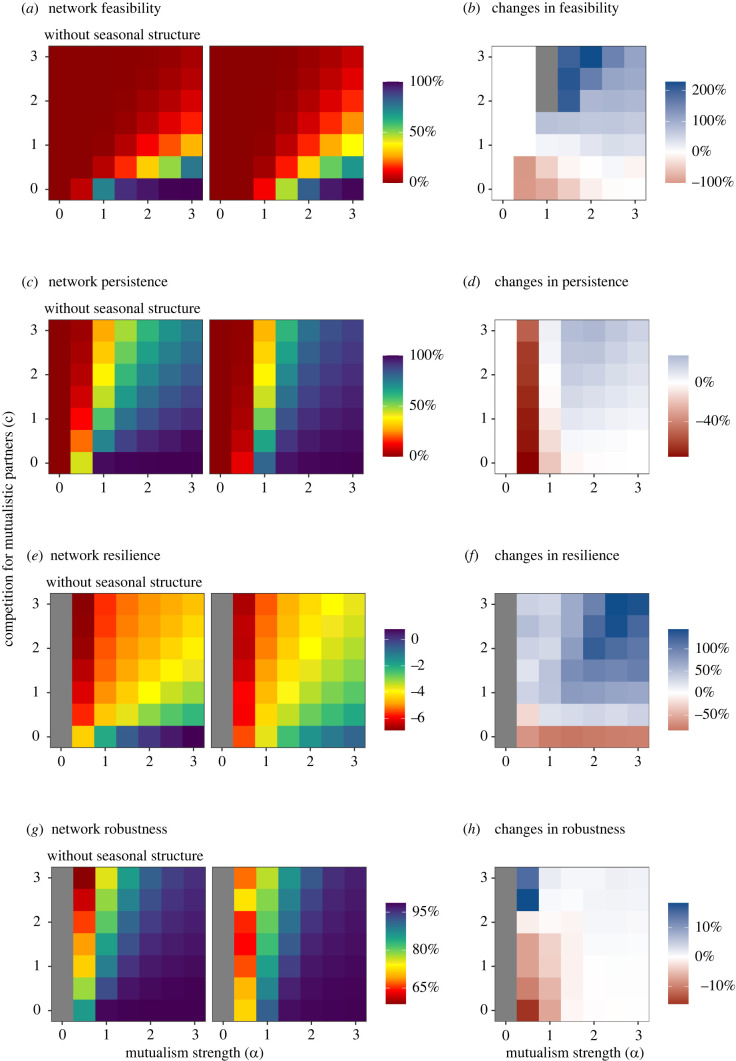
Effect of the seasonal structure of interactions on stability metrics as a function of competition and mutualism strengths, averaged over the 11 communities. (*a,c,e,g*) show values of stability metrics, without or with seasonal structure, while (*b,d,f,h*) represents relative changes in stability metrics between the two sets of simulations, without and with the seasonal structure. Relative changes can be high even when absolute differences are low, because they are expressed in percentage (±%) relative to the baseline ‘without seasonal structure’ for a given combination of *α* and c, to compare effects on stability measures over parameter combinations. Values of (*a*) network feasibility (percentage of simulations with more than 98% of persisting species), (*c*) network persistence (the percentage of persistent species at the equilibrium), (*e*) network resilience (invert of the time to return at the equilibrium after a small perturbation), (*g*) network robustness, percentage of species of a community surviving to the extinction of a given species, without (left) or with (right) including the seasonal structure of interactions. Values are averaged over the 11 communities and simulations for each combination of competition (*c*) and mutualism (*α*) strengths. Grey cells represent cells without values, either because network persistence equals zero either because relative changes are not defined (impossible division by zero). In (*e–h*) we used only networks with a non-null equilibrium (*n* = 182 901), i.e. with at least two species, since mutualism is obligatory. (Online version in colour.)

First, all metrics of network stability strongly increase with mutualism strength and strongly decrease with the strength of competition for mutualistic partners (figure 2*a,c,e,g*). This result is expected and consistent with previous studies [19,20,24]; the higher the ratio *α*/*c* is, greater are the benefits of mutualistic interactions for species (electronic supplementary material, figure S3). Here, greater benefit from mutualistic interactions stabilize communities because we used a saturating functional response, to account for the fact that interaction rates cannot be infinite because mutualistic interactions have a certain handling time (i.e. pollinator looking for the flower, entering in the flower and leaving). This result does not necessary hold if using a linear functional response because a high mutualism strength can destabilize communities through infinite population growth due to artefactual orgies of mutual benefaction [25].

More importantly for our question, our results show that the effect of the seasonal structure of interactions also strongly depends on the strength of competition for mutualistic partners and mutualism (figure 2). Since results across the 11 communities were highly consistent, we present averaged results, but community-level results are available in electronic supplementary material, figures S4–S7. Since we modelled obligate mutualism (i.e. species with negative growth rates *r_i_*_/*j*_), when the mutualism strength is null (*α* = 0) no species survive and the network feasibility and persistence equal zero (figure 2*a,c*). When mutualism strength is weak (*α* = 0.5), seasonal structure decreases the network feasibility and persistence, because accounting for the phenological overlaps, which vary between 0 and 1, weakens plant–hummingbird interactions, which are then not strong enough to support persistence of many species. As expected from analytical analyses (cf*.* electronic supplementary material, 'Methods', §S2), when neither mutualism nor competitions for mutualistic partners are weak (*α* and *c* > 0.5), then seasonal structure increases network feasibility (figure 2*b*), i.e. the probability to reach an equilibrium including greater than 98% of species observed in given community. This result is due to the fact that seasonal structure generally extends the feasibility domain to more negative growth rates for plants, allowing the full community to persist even when plants are strongly dependent on hummingbirds (electronic supplementary material, figure S8).

Seasonal structure affects network persistence in a similar way, increasing persistence in a competition context when mutualism strength is high enough to support viable species populations (figure 2*c,d*). Seasonal structure also increases resilience when we include competition for mutualistic partners in simulations (*c* > 0; figure 2*e,f*), suggesting that the ecological equilibriums reached in simulations are more diverse and more resilient when including seasonal structure. By contrast, seasonal structure does not affect network robustness (figure 2*h–g*), except when the mutualism strength is low (*α* ≤ 1); in this case seasonal structure strongly decreases network robustness.

The effects of seasonal structure on network stability are much more positive when considering empirical seasonal structures as compared to randomized seasonal structures (electronic supplementary material, figure S9) that have the same backbone as empirical ones (same binary interaction matrix *I*; see 'Methods'). This result suggests that it is the specific way interactions are organized along the season in empirical communities which increases feasibility, persistence and resilience.

**Figure 3.**
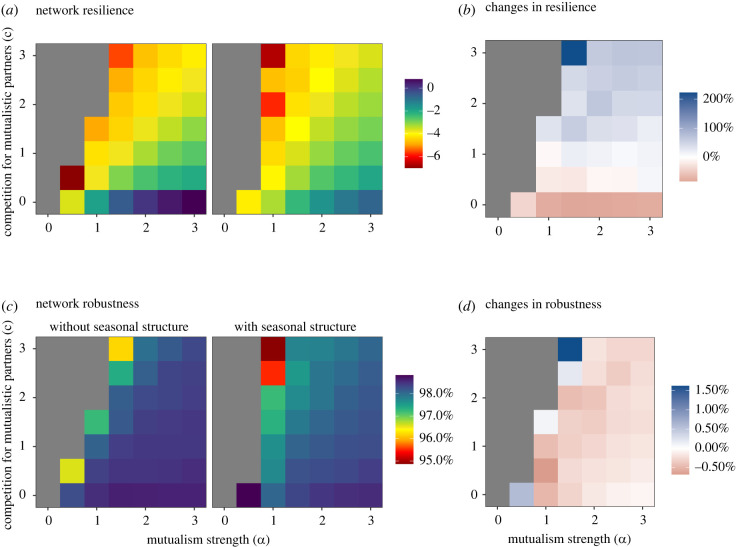
Effect of seasonal structure on stability metrics as a function of competition and mutualism strengths, considering feasible equilibriums only. (*a,c*) show values of stability metrics, without or with seasonal structure, while (*b,d*) represents relative changes in stability metrics between the two sets of simulations, without and with seasonal structure, in percentage relative to the simulations without seasonal structure. (*a–f*) are the same as figure 2*c–f*, but now only feasible equilibriums are considered (*n* = 42 218). (*a*) network resilience, and (*c*) network robustness, without (left) or with (right) including the seasonal structure of interactions. Values are averaged over the 11 communities and simulations for each combination of competition (*c*) and mutualism (*α*) strengths. Grey cells represent cells without values. Note that colour scales change among panels. (Online version in colour.)

At equilibrium, diversity strongly varies among communities, depending on the values of *α* and *c* (figure 2*c*; i.e. persistence is a measure of diversity at equilibrium, relative to initial conditions). Since resilience and robustness depend on diversity at equilibrium, interpretation of figure 2*e–h* is difficult, as the patterns could be driven by changes in diversity at equilibrium. To overcome this issue, we focused on feasible ecological equilibriums, those maintaining more than 98% of the species present in each community, which thus vary only minimally in persistence and diversity at equilibrium. We find that resilience is still positively affected by seasonal structure while robustness remains almost unaffected (figure 3), suggesting that results observed in figure 2*f,h* are not due to variation in persistence.

**Figure 4.**
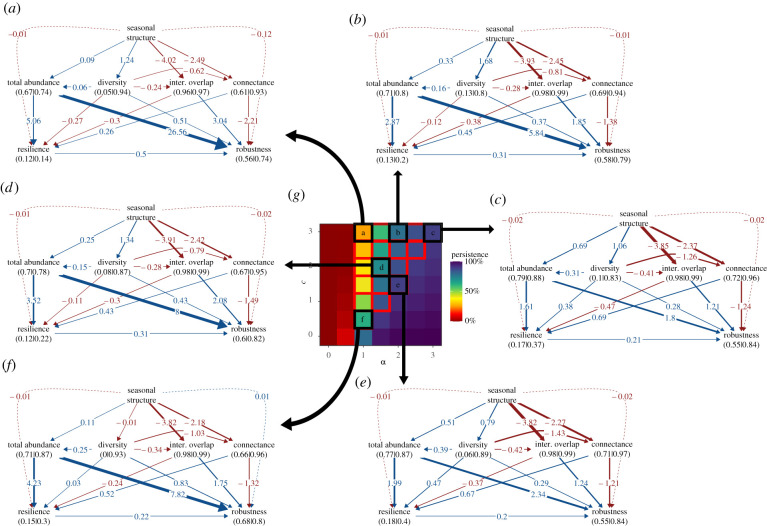
Effects of the seasonal structure on network indices and network stability as a function of competition and mutualism strengths. (*a–f*) show path-analyses for six different combinations of competition (*c*) and mutualism (*α*) strengths. Solid lines and numbers correspond to either positive (blue) or negative (red) standardized effects, comparable among path analyses, while dashed lines represent residual effects with a similar colour code (see 'Methods'). Left numbers under the variable names are the marginal r-squared values, while right ones are the conditional *r*-squared values. (*g*) represents network persistence with seasonal structure, it is the same as figure 2*a* right; cells highlighted in red correspond to the combinations of parameters in which the average network persistence is between 20% and 90%, allowing to disentangle effects of studied variables while avoiding too high collinearity among variables (see 'Methods'). Cells highlighted in black correspond to the combinations of parameters for which we show the path analyses in (*a–f*). In (*a–g*) we used only networks with a non-null equilibrium, so with at least 2 species, since mutualism is obligatory. Inter. overlap: interaction overlap. All relationships presented in (*a–e*) are significant (*p*-value < 0.05), excepting those presented in dashed arrows (residual effects). (Online version in colour.)

Seasonal structure can affect network stability by driving changes in variables describing network structure, including diversity (number of species at equilibrium), connectance between guilds at equilibrium, interaction overlap within guilds at equilibrium and total abundance (the sum of species abundances at equilibrium) (electronic supplementary material, figure S10). To disentangle which of these network variables mediate the effects of seasonal structure on resilience and robustness, we used path analyses. For each parameter combination (i.e. values of *α* and *c*) that lead to intermediate levels of persistence on average (20% < persistence < 90%; see 'Methods'), we performed a path analysis on the 5500 simulations corresponding to this parameter combination (figure 4).

Path analyses highlight the important role of the four tested network variables in mediating effects of seasonal structure on network stability (figure 4). First of all, seasonal structure, through the fact that it increases total abundance, strongly increases network stability, measured as resilience and robustness (figure 4*a–f*). For resilience, this result is likely because of the positive relationship between the speed of population dynamics and species abundance: species with high abundance return more quickly to the equilibrium after a small perturbation than do rare species. For robustness, this result is because high total abundance means that on average, species are abundant, and thus are less likely to go extinct than rare species that have abundance values close to the extinction threshold.

Second, seasonal structure, through the fact that it decreases interaction overlap within guilds, has contrasting effects on network stability, increasing resilience but decreasing robustness (figure 4*a–f*). Decreasing interaction overlap within guilds creates more modular interaction networks, restricting the propagation of a perturbation within a module, thus increasing resilience. However, as the interaction overlap within guilds (i.e. functional redundancy) decreases, the probability that other species can buffer the extinction of a given species also decreases, resulting in lower robustness. In other words, decreasing interaction overlap restricts perturbation propagation within the module containing the perturbed species, but increases the sensitivity of this module to the perturbation.

Third, seasonal structure, through the fact that it decreases the connectance among plants and hummingbirds, increases robustness but decreases resilience (figure 4*a–f*). A decrease in resilience results because seasonal structure yields fewer links between hummingbirds and plants, which increases their dependency on each other, in turn, increasing the effect of small perturbations. The increase in robustness results because decreasing connectance decreases the probability that the extinction of a given species affects many other species.

Finally, seasonal structure, through the fact that it increases diversity at equilibrium, affects resilience in a complex way and increases robustness. For resilience, the direction of this diversity-mediated effect depends on the strengths of competition and mutualism (figure 4*a–f*), mainly because the diversity–resilience relationship shifts from negative, when persistence is low, to positive, when persistence is high (figure 4*a–f*). This shift is likely because in a system with few species, maintaining more species will decrease resilience because it increases the chance of a species with slower population dynamics, slowing the return of a community to equilibrium after a small perturbation. By contrast, when considering diverse systems, maintaining more species decreases the effect of small perturbations, thus increasing resilience. For robustness, more diverse systems lead to a weaker dependency on specific mutualistic partners, then decreasing the number of secondary extinctions following the extinction of a given species.

By multiplying the standardized effects along paths linking seasonal structure to resilience or robustness, shown in figure 4*a–f*, we can assess the importance of network variables in mediating the effects of seasonal structure on stability. We show that effects of seasonal structure on resilience and robustness are mainly mediated by network structure (i.e. connectance and interaction overlap) and total abundance, while diversity does not play a major role (electronic supplementary material, figure S12). Moreover, direct effects of diversity on resilience and robustness are weak and often less important than indirect effects, through total abundance, connectance and interaction overlap (electronic supplementary material, figure S12a). Finally, an important part of the effect of diversity on resilience is mediated by the total abundance at equilibrium (i.e. productivity), while abundance is less important for robustness (electronic supplementary material, figure S12b).

## 3. Discussion

Our results provide support of the positive effect of the seasonal structure on mutualistic network persistence, resilience and feasibility. We extended previous theoretical findings, which were obtained on simulated seasonal structures [15,17], by using the empirical seasonal structure from 11 sampled plant–hummingbird communities. Assuming that species compete for mutualistic partners, we find that when mutualism strength is strong enough to support viable species populations, seasonal structure of interactions enhances network persistence and resilience, allowing more diverse and resilient ecological equilibriums than without seasonal structure, while maintaining the network robustness and extending the feasibility domain. The fact that empirical seasonal structures have much more positive effects on persistence and resilience than randomized seasonal structures, suggests that eco-evolutionary dynamics occurring in nature led to a seasonal organization of interactions and species which promote community stability.

The simulations without seasonal structure allows us to place our model in a broader context. By contrast to analytical analyses of simpler models [26], we find that decreasing the competition for mutualistic partners and increasing the mutualism strength increases all studied facets of stability, namely, feasibility, persistence, resilience and robustness. However, our results are in line with numerical simulations including a saturating term in the functional response (i.e. positive handling time, *β*), ensuring stability when the strength of mutualism is high [20,24].

The simulations with seasonal structure show that seasonal structure increases network stability only when competition for mutualistic partners is non-negligible. This is consistent with previous theoretical results showing that the seasonal structure of interactions increases community persistence by decreasing competition [16,27] and promoting facilitation over competition in mutualistic networks [17]. Our simulations also show that seasonal structure increases the likelihood of reaching a feasible equilibrium, thus extending the set of environmental conditions in which species can coexist.

We found that network resilience and robustness are strongly determined by the total abundance at equilibrium, the connectance among guilds, and the interaction overlap within guilds, a measure of effective competition, while diversity does not play a major role. These results are consistent with the theoretical findings of Pascual-García & Bastolla [20] on network resilience, showing that in a case of obligatory and saturating mutualism, resilience is positively linked to network connectance and negatively linked to effective competition. Moreover, we found that interaction overlap within guilds strongly determine, sometimes even more than connectance among guilds, network resilience and robustness. These findings suggest that competition structure is as important as the structure of mutualistic interactions for predicting network stability, which echoes recent experimental findings showing that effective competition among plants and among pollinators strongly determines community stability [28], and stresses the need to develop more multitrophic experimental studies.

Regarding robustness, pioneering studies have shown that robustness of mutualistic network depends on the phenological attributes of the species removed [29] or on the season [13,14]. Our study uses a different angle, estimating the robustness of the overall networks with or without accounting for the seasonal structure, instead of estimating how robustness of a given network changes over the seasons. We show that the seasonal structure of interactions tends to weakly affect the robustness of the overall network, as soon as the mutualism is not too weak (*α* > 0.5). This result is due to the fact that seasonal structure affects network robustness in contrasting ways through different paths. By decreasing the functional redundancy at a given moment (i.e. interaction overlap), seasonal structure makes the overall network more sensitive to species extinction, but by decreasing the connectance between guilds, it increases the robustness, because it prevents further extinctions [30]. So, the multiple paths through which seasonal structure affects network robustness could make it hard to predict the consequences of current phenological shifts in plant flowering or pollinator activity on the robustness of communities to extinctions. In addition to affect interaction overlap [31–34], phenological shifts will probably also affect the connectance and the productivity (i.e. total abundance) of communities, which also determine network robustness.

Here we also show that independent to its effect on diversity, seasonal structure promotes community productivity, which has strong positive effects on network resilience and robustness. Thus, in addition to playing an important role in community stability, the seasonal structure of interactions also plays an important role in enhancing ecological functions, suggesting that it could be important for understanding the diversity–productivity relationships. In addition, most of the effects of seasonal structure on resilience or robustness that appear to be mediated by diversity are, in fact, only indirectly mediated by diversity and involve either productivity or network structure. This result suggests that holistic approaches tackling diversity–stability and diversity–productivity debates together could yield further insights on the topic.

Since our results are based on theoretical models, they rely on strong assumptions which can limit the validity of our results. However, using supplementary simulations we show that the positive effect of seasonal structure on feasibility, persistence and robustness, described above, still hold when important assumptions are relaxed: relaxing the assumption of obligatory mutualism by introducing plants that do not completely depend on hummingbird for reproduction (electronic supplementary material, figure S13), and adding a new kind of competition to the model, that is interspecific competition for resources independent from mutualistic interactions (space, light, breeding place, etc.; electronic supplementary material, figure S14). Supplemental analyses suggest that our results are valid even outside of the tested range of *α* and *c* (see electronic supplementary material, 'Methods' and figure S15), however since we took advantage of numerical simulations to explore seasonal dynamics of diverse communities, fully exploring the parameter domain was impossible, as some parameters do not have natural bounds (e.g. growth rates can be defined on (−∞,+∞)). Analytical analyses of simpler models could help to fully assess the generality of our results, but these would necessarily involve fewer species or a simpler functional response, making the theoretical models even more abstract. We did however, avoid excessive complexity of our model by not including any density-dependent behaviour, such as adaptive foraging. Nonetheless, empirical data [35] and models [4] suggest that adaptive foraging determines interaction rewiring over seasons and affects network stability, stressing the need to jointly model adaptive foraging and seasonal dynamics to study their interactions and consequences for network stability.

Although our approach is based on an unavoidable simplification of natural systems, it provides new insights into the role of seasonal structure of interactions in promoting the stable coexistence of a diverse set of hummingbirds and plants. Recent theoretical and empirical findings showing similar results on persistence of food webs [36,37] and competitive networks [16,27,38] suggest that the seasonal structure of species interactions is a common stabilizing mechanism of natural communities. Thus, the phenological shifts, driven by climate change, which are changing the seasonal distribution of species [31,32,39–41] could widely affect stability of natural communities.

## 4. Methods

Our goal was to study the effect of the seasonal structure of plant–pollinator interactions on stability of plant–pollinator interaction networks. To do so, we used an empirical dataset of plant–hummingbird interaction networks sampled all along the year, to parameterize a dynamic model, accounting or not for the seasonal structure of interactions, i.e. the monthly structure in interactions (figure 1). Using this model, we assessed the effect of this seasonal structure on four measure of network stability: feasibility, persistence, resilience and robustness.

### (a) Empirical dataset of interactions

We used data from 11 independent sites in the tropical forests of Ecuador (table 1; electronic supplementary material, figure S1) in which interactions among flowering plants and hummingbirds were recorded along transects by using camera traps, as described in 2018 [42]. Sites were sampled between 2013 to 2021 with an average of 4.72 ± 2.83 (mean ± s.d.) years of sampling and on average 10.36 ± 0.87 sampled months per year and per site. Here, the several years of sampling are aggregated per month, considering, for example, December 2013–2021 as replicates of the same thing.

**Table 1.** Community descriptions.

site name	average elevation (m)	plant richness	hummingbird richness	number of unique interactions	first year of sampling	last year of sampling
Alaspungo	2878	40	7	113	2018	2019
Las Gralarias	2051	59	13	163	2017	2019
Maquipucuna	1604	65	17	238	2013	2021
MashpiCapuchin	930	43	15	100	2017	2019
MashpiLaguna	1189	41	17	126	2017	2019
Sachatamia	1685	44	10	125	2017	2019
Santa Lucia Lower	1946	71	22	221	2013	2021
Santa Lucia Upper	2298	55	14	235	2013	2021
Un Poco del Choco	1163	46	13	130	2017	2021
Verdecocha	3383	46	14	138	2017	2019
Yanacocha	3510	32	11	101	2017	2019

### (b) Interaction matrices and seasonal structures

We build an interaction network and a binary interaction matrix *I* for each of the 11 communities (electronic supplementary material, figure S2), where $I_{ij}$ describes the occurrence of interaction between hummingbird species *j* (*j*th column) and plant species *i* (*i*th row). $I_{ij}$ equals one if two species were observed to interact at least once in a given community, and equals zero otherwise. For each community, this interaction matrix was the backbone of our theoretical model described below. Since communities were sampled monthly, we could split the interaction networks in monthly sub-networks (figure 1) and extract a set of indices characterizing phenological overlap in interaction for each pair of species, among guilds (between plants and hummingbirds) or within guilds (plant or hummingbird). This set of indices described the seasonal structure, which corresponds to the way plant–hummingbird interactions are organized along the season. First, we calculated phenological overlap ($O_{ij}$) between hummingbird species *j* and plant species *i* for each pair of species (see electronic supplementary material, 'Methods', §S1). Thus, hereafter, we assumed that a plant and a hummingbird are able to interact only if at least one interaction between them was recorded before, and that the interaction strength *per capita* will be proportional to their phenological overlap.

Second, we calculated the phenological overlap among pairs of species from the same guild. Since we wanted to know how these species co-occur in the same plant at the same time, to use this index as a proxy of competition interaction (cf*.* below), we calculated this index per mutualistic partner. For hummingbirds, we calculated $M_{h_{ikj}}$, which is the phenological overlap among hummingbirds *k* and *j* on plant *i*; while for plants, we calculated $M_{\;p_{\;jki}}$, which is the phenological overlap among plants *k* and *i* for hummingbird *j*. $M_{h_{ikj}}$ and $M_{\;p_{\;jki}}$ were calculated as follow:

4.1 $$M_{h_{ikj}}=\overset{12}{\underset{m=1}{\sum }}\frac{F_{m_{i}}}{\sum_{m=1}^{12}F_{m_{i}}}\times \frac{F_{m_{k}}}{\max(F_{m=1\ldots 12_{k}})}\;\times \frac{F_{m_{j}}}{\max(F_{m=1\ldots 12_{j}})}$$

and

4.2 $$M_{\;p_{\;jki}}=\overset{12}{\underset{m=1}{\sum }}\frac{F_{m_{j}}}{\max(F_{m=1\ldots 12_{j}})}\;\times \frac{F_{m_{k}}}{\sum_{m=1}^{12}F_{m_{k}}}\times \frac{F_{m_{i}}}{\sum_{m=1}^{12}F_{m_{i}}},$$

where $F_{m_{j}}$ ($F_{m_{i}}$) is the average number of interaction of hummingbird *j* (plant *i*) in month *m*. $M_{h_{ikj}}$ is the term of a $M_{h}$ array of dimensions $n_{p}\times n_{h}\times n_{h}$, while $M_{\;p_{\;jki}}$ is the term of a $M_{p}$ array of dimensions $n_{h}\times n_{p}\times n_{p}$.

### (c) A theoretical model

We used a theoretical model, largely based on the one used in Duchenne *et al.* [17], describing the interactions between two guilds, hummingbirds (*H*) and plants (*P*). Species belonging to the same guild compete with each other for partners, and species from distinct guilds interact mutualistically. We modelled the dynamics of the abundance of birds for each hummingbird species and of flowers for each plant species, for each community using the following equations:

4.3 $$\frac{dH_{j}}{dt}=H_{j}(r_{j}+\frac{\alpha \sum_{i=1}^{n_{p}}I_{ij}\times O_{ij}\times P_{i}}{1+\beta \sum_{i=1}^{n_{p}}I_{ij}\times O_{ij}\times P_{i}+c\sum_{k=1}^{n_{h}}\Omega_{kj}\times H_{k}}-\overset{n_{h}}{\underset{k=1}{\sum }}c_{s_{\;j,k}}\times H_{k})$$

and

4.4 $$\frac{dP_{i}}{dt}=P_{i}\left(r_{i}+\frac{\alpha \sum_{\;j=1}^{n_{h}}I_{ij}\times O_{ij}\times H_{j}}{1+\beta \sum_{\;j=1}^{n_{h}}I_{ij}\times O_{ij}\times H_{j}+c\sum_{k=1}^{n_{p}}\Theta_{ki}\times P_{k}}-\overset{n_{p}}{\underset{k=1}{\sum }}c_{s_{i,k}}\times P_{k}\right)$$

*H_j_* corresponds to the number of individual pollinator species *j* and *P_i_* to the total number of flowers produced by plant species *i* over one year. We can split the between brackets part of equations (4.3) and (4.4) in three parts: growth rates, benefits of mutualism (including competition for mutualistic partners) and intraspecific competition for space.

The first one is the basal growth rate, which correspond to the growth rate of this species when not accounting for any inter or intra-specific interactions. $r_{j}$ is the growth rate of the hummingbird species *j*, while $r_{i}$ is the growth rate of the plant species *i*. We assumed negative growth rates for plants and hummingbirds, thus assuming obligate mutualisms between both guilds.

The second part models the benefits of mutualism on the population growth of plant and hummingbird species by a functional response which depends both on the abundance of the mutualistic partners and abundance of the within-guild competitors. The mutualism benefit for species *j* increases with α, which is the mutualism strength between guilds, and with the interaction strength per capita of species *j* with its mutualistic partners $I_{ij}\times O_{ij}$. Second, the benefit saturates with the abundance of the mutualistic partners depending on the handling time parameter β. Third, it decreases with the abundance of within-guild competitors depending on the maximum competition strength *c*, as detailed below.

The third part of the equation corresponds to competition for space, or whatever else is independent from competition for mutualistic partners. This intra-specific competition has a strength of one for both, plants ($c_{s_{i,k=i}}=1$) and hummingbirds ($c_{s_{\;j,k=j}}=1$). For simplicity, here we do not consider interspecific competition independent from mutualistic partners, thus setting $c_{s_{i,k\neq i}}=0$ and $c_{s_{\;j,k\neq j}}=0$.

Going back to the second part of equation (4.3), corresponding to the competition for mutualistic partners, $\Omega_{kj}$ is the competition coefficient imposed by hummingbird *k* on the focal hummingbird species *j*, thus if k=j it is intra-specific competition, otherwise it is interspecific competition. These competition coefficients vary from 0 (no competition) to 1 (maximal competition) and increase if species share common mutualistic partners at the same time. Those competition coefficients were calculated for each pair of hummingbird species and stored in a $n_{h}\times n_{h}$ matrix Ω, and for each pair of plant species and stored in an analogue matrix called Θ. $\Omega_{kj}$ and $\Theta_{ki}$ are calculated using analogue equations:

4.5 $$\Omega_{kj}=\frac{1}{\sum_{i=1}^{n_{p}}I_{ij}\times P_{i}\times O_{ij}}\times \overset{n_{p}}{\underset{i=1}{\sum }}P_{i}\times I_{ij}\times I_{ik}\times M_{h_{ikj}}$$

and

4.6 $$\Theta_{ki}=\frac{1}{\sum_{\;j=1}^{n_{h}}I_{ij}\times H_{j}\times O_{ij}}\times \overset{n_{h}}{\underset{\;j=1}{\sum }}H_{j}\times I_{ij}\times I_{kj}\times M_{\;p_{\;jki}},$$

where $M_{h_{ikj}}$ is the phenological overlap among hummingbirds *k* and *j* on plant *i*; while $M_{\;p_{\;jki}}$ is the phenological overlap among plants *k* and *i* for hummingbird *j*.

Thus, *O*, $M_{h}$ and $M_{p}$ are the only elements of the model that include information about the seasonal structure of interactions. When we wanted to remove the seasonal structure in the model, we set all terms of these matrices to 1.

### (d) Numerical simulations

To solve the model, we fixed arbitrary values for some parameter values as well as for initial abundances for $H_{j}$ and $P_{i}$, using given values outlined in table 2. Growth rates which were drawn randomly from a beta distribution (*B*), to sample a wide variety of possible vectors of growth rates. We fixed the first shape parameter at a=1 and we drew the second shape parameter *b* from $e^{U(\log(0.3),\log(15))}$ to sample values between 0.3 and 15, but with more *b* values around 0.3 than around 15, allowing to model a wide diversity of growth rate distributions (*cf.* electronic supplementary material, figure S4b). To avoid that communities collapse too often, we bounded the space of growth rates between −0.5 and 0 by dividing by two the values drawn in the beta distribution. The growth rates of plants and hummingbirds were drawn independently from this equation:

4.7 $$r_{i/j}∼\frac{-1}{2}\times B(a=1,b∼e^{U(\log(0.3),\log(15))})$$

**Table 2.** Parameter values of the dynamic model. Parameter combinations correspond to the different combinations of strength of competition for mutualistic partner and mutualism strength, which are the parameters of interest here. Growth rates vary among species and among the 250 initial conditions in order to explore a wide set of possible cases.

parameter	meaning	value	variation among		
			species	250 initial conditions	parameter combinations
p*_i_*/h*_j_*	plant/hummingbird initial abundances	1	no	no	no
*α*	mutualism strength	0/0.5/1/1.5/2/2.5/3	—	no	yes
*c*	competition strength for mutualistic partners	0/0.5/1/1.5/2/2.5/3	—	no	yes
*β*	handling time	0.8	no	no	no
*r_i_*_/*j*_	plant/hummingbird growth rates	equation (4.7)	yes	yes	no
$c_{s_{i/j,k=i/j}}$	intra-specific competition within guilds for space	1	no	no	no
$c_{s_{i/j,k\neq i/j}}$	interspecific competition within guilds for space	0	no	no	no

We considered each community as independent, and we created 250 different initial conditions for each community. For each of these initial conditions we solved the model using 7 values of mutualism strength, between 0 and 3, and 7 values of competition strength, between 0 and 3 (table 2), but results are expected to be robust to other values of competition strength (*c*) and mutualism strength (*α*), as detailed in electronic supplementary material, Methods 2. We performed two sets of simulations, one considering the seasonal structure of interactions, the other one neglecting it (setting all the elements of *O*, $M_{h}$ and $M_{p}$ to one), that resulted in 269 500 simulations (11 × 250 × 7 × 7 × 2).

We re-performed these simulations but randomizing among species the monthly counts of interactions that were positive, $F_{m_{i}}$ and $F_{m_{j}}$, independently for plant and hummingbirds for each of the 250 initial conditions. This manipulation allowed creation of randomized seasonal structures, but with the same backbone as the empirical ones, so without changing the binary interaction matrices and without breaking existing interactions, to test if the effects of the empirical seasonal structure on the stability differ from randomized seasonal structures.

Finally, to see how our results were affected by model assumption, we re-performed two sets of simulations (figures S13 and S14) changing either: (i) the plant growth rate (*r_i_ -* *>* *r_i_ – median(r_i_)*) or (ii) the plant growth rates (*r_i_ -* *>* |*r_i_*|) and interspecific competition for space ($c_{s_{i/j,k\neq i/j}}=0.05$). The first set of simulations released the assumption that mutualism is obligatory by making 50% of plants partially independent of hummingbirds (i.e. positive growth rates). The second set of simulations assumed that all plants are partially independent from hummingbirds, but all compete together for another resource (space, light, etc.) in addition to compete for hummingbirds.

### (e) Network stability

We evaluated four network stability metrics at the equilibrium: feasibility, persistence, resilience and robustness.

Feasibility is often assessed using analytical methods [12,24], but considering the complexity of our model such kind of approach is not applicable. Thus, we assessed the feasibility using simulations over a wide diversity of growth rates (cf*.* above), as the percentage of simulations with a network persistence > 98%. We defined feasibility with this threshold, and not as persistence equals to 100%, to allow the loss of one species in diverse community (>50 species) and zero otherwise. This is because these diverse communities are likely to lose species because of imperfect interaction sampling and because our system probably consists of open instead of closed populations.

Network persistence was measured as the percentage of persisting species at equilibrium, with an abundance greater than 10^−5^ at the equilibrium, other species being considered as extinct, for each community and simulation. When persistence was expressed in a number of species present at equilibrium instead of percentage of persisting species, it was called diversity.

Network resilience was evaluated as −max(Re(λ)), where λ are the eigenvalues of the Jacobian matrix of our system, after removing extinct species. Resilience is inversely proportional to the time to return to the equilibrium τ∼(1/−max( Re(λ))).

Network robustness was measured as the number of secondary extinctions following the removal of a species. To save computational time, we only removed hummingbird species but assess the robustness over both guilds, as detailed in electronic supplementary material, Methods.

### (f) Network indices

We evaluated the connectance of the network as an index of network structure at equilibrium. We measured this index on the network matrix (*N*), which is the interaction matrix corrected by the phenological overlap (N=I×O), after removing extinct species. The connectance is the mean of all the terms of *N*. In addition, since our species are also linked within guilds through the competition for mutualistic partner, we also evaluated an index of the connectance within guilds, which is the average interaction overlap among species from the same guild, at the equilibrium. This index is the mean of all terms of Ω and Θ, when all species abundances are set to 1 and after removing extinct species. Finally, we calculated the total abundance of the community, which is the sum of hummingbird and flower abundances over all species present and the given community, for the given simulation. This total abundance was considered as a measure of productivity.

### (g) Statistical analyses

Since the implementation of the seasonal structure of interactions will affect indices of network structure (connectance and interaction overlap) and is susceptible to affect total abundance and diversity at equilibrium, we used a path analysis to disentangle which variables mediate effects of the seasonal structure on resilience and robustness. We tested all links presented in figure 4, as detailed in electronic supplementary material, 'Methods'.

We performed this path analysis for each combination of competition and mutualism strengths that do not lead to high collinearity among variables. To solve collinearity pissues we excluded combinations of competition and mutualism strengths that allow the persistence of greater than or equal to 90% of the species on average, with and without seasonal structure, and to avoid to focus on communities with very low diversity we also excluded parameter combination leading to network persistence less than 20% on average. We also excluded the simulations without competition for mutualistic partners (*c* = 0), as this is a control situation, and networks with a persistence equals to zero as resilience and robustness could not be estimated. However, as the number of parameter combinations for which we performed path analyses is high (*n* = 18) we presented results only for six different combination of mutualism and competition strengths (figure 4).

## Data Availability

Data are available from the Dryad Digital Repository (https://doi.org/10.5061/dryad.vhhmgqnvw) [43]. Code is available here: https://github.com/f-duchenne/Seasonal-structure-enhances-stability-of-mutualistic-networks. Electronic supplementary material is available online [44].
